# Effect of Porosity on Dynamic Mechanical Properties and Impact Response Characteristics of High Aluminum Content PTFE/Al Energetic Materials

**DOI:** 10.3390/ma13010140

**Published:** 2019-12-30

**Authors:** Chunlan Jiang, Shangye Cai, Liang Mao, Zaicheng Wang

**Affiliations:** State Key Laboratory of Explosion Science and Technology, Beijing Institute of Technology, Beijing 100081, China; jiangchunwh@bit.edu.cn (C.J.); 3120170154@bit.edu.cn (S.C.); wangskyshark@bit.edu.cn (Z.W.)

**Keywords:** energetic materials, porosity, dynamic mechanical properties, impact response

## Abstract

In order to obtain the effect of porosity on the dynamic mechanical properties and impact response characteristics of high aluminum content PTFE/Al energetic materials, PTFE/Al specimens with porosities of 1.2%, 10%, 20%, and 30% were prepared by adding additives. The dynamic compression properties and impact response characteristics of high aluminum content PTFE/Al energetic materials with porosity were studied by using a split Hopkinson pressure bar (SHPB) impact loading experimental system. Based on the one-dimensional viscoplastic hole collapse model, an impact temperature rise analysis model including melting effects was used, and corresponding calculation analysis was performed. The results show that with the increase of porosity, the yield strength and compressive strength of the material will decrease. Under dynamic loading, the reaction duration of PTFE/Al energetic materials with different porosities generally shows a tendency to become shorter as the porosity increases, while the ignition delay time is basically unchanged. In this experiment, the material response has the optimal porosity with the lowest critical strain rate, the optimal porosity for PTFE/Al energetic materials with different porosity and high aluminum content (50/50 mass ratio, size of specimens Φ8 × 5 mm) is 10%. The research results can provide an important reference for the engineering application of PTFE/Al energetic materials.

## 1. Introduction

Metal and fluoropolymer composites are energetic materials that can initiate chemical reactions when impacted. This material is inert in the general environment [1], but under impact loading conditions, the impact energy will cause a severe chemical reaction between the active metal and the fluoropolymer, forming a detonation-like effect. PTFE/Al is one of the research hotspots of such metal and fluoropolymer energetic materials, and high aluminum content PTFE/Al energetic materials have broader application prospects as warhead damage metamaterials due to their higher material density and higher sound velocity.

Previous researchers have carried out much research work on the component ratio, particle size, density, and material reaction characteristics of PTFE-based energetic materials. Shen [2] used Taylor bar impact experiments to study the law of deformation and ignition time of PTFE/Ti energetic materials caused by the impact of the material ratio and the impact speed of the bar. Wu et al. [3] used drop weight experiments to characterize the reaction characteristics of PTFE/Al and concluded that the characteristic drop height and ignition energy increase with the increase of Al particle size. McGregor et al. [4] studied the impact response threshold of 40% density PTFE/Al energetic materials under impact. Dolgoborodov et al. [5] found that porous PTFE/Al (0.4~0.5 g/cm^3^) energetic materials can form stable detonation under the impact pressure of 1 GPa. By testing the reaction process of metal fluorocarbon-based reaction materials, Koch et al. [6] concluded that the material’s ability to react in metal fluorocarbon-based systems is significantly correlated with temperature. Feng et al. [7] carried out a quasi-static compression experiment of PTFE/Al energetic materials and proposed a PTFE/Al crack-induced initiation mechanism based on experimental phenomena. At the same time, porosity, as an important consideration in the preparation of materials, will have an important impact on the reaction threshold and reaction time of the material.

In view of the previous studies on the effects of porosity on the dynamic mechanical properties and impact response characteristics of PTFE/Al energetic materials, there is little research, especially for high aluminum content PTFE/Al (mass ratio 50/50) energetic materials. In this paper, a split Hopkinson pressure bar (SHPB) experimental test system will be used to conduct research on the effect of porosity on the dynamic mechanical properties and reaction characteristics of high aluminum content PTFE/Al energetic materials, in order to provide an important reference for the engineering application of high aluminum content PTFE/Al energetic materials.

## 2. Experimental Methods

### 2.1. Material Preparation

In order to obtain PTFE/Al energetic materials with different porosity, the wet-mixing process based on the traditional compression sintering preparation [8] was improved, the additive sintering volatility characteristics to prepare pore containing materials were used. First, using a small amount of absolute ethanol as the medium, PTFE powder, Al powder, and additives (polypropylene powder) were placed in a mixer to wet-mix evenly, and then the mixed powder was put in a drying box to dry it. After the body dried, the mixed powder was pressed into a mold by uniaxial cold pressing. Finally, the blank was placed in a sintering furnace and sintered in an argon atmosphere. The related properties of polypropylene (PP) powder are shown in Table 1. Its melting point is lower than the sintering molding temperature, and it will volatilize during sintering [9].

### 2.2. Material Properties

**Figure 1:**
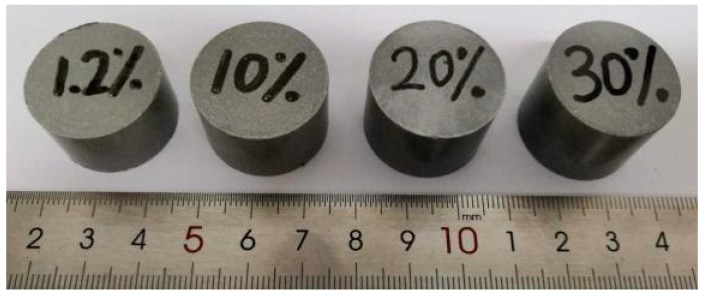
Specimens of PTFE/Al with different porosity.

Figure 1 shows PTFE/Al specimens prepared with porosities of 1.2%, 10%, 20%, and 30%, respectively. Table 2 lists the masses before sintering, the masses after sintering, the balance masses before and after sintering, the masses of additives, and the measured porosity of four different PTFE/Al energetic materials.

**Figure 2:**
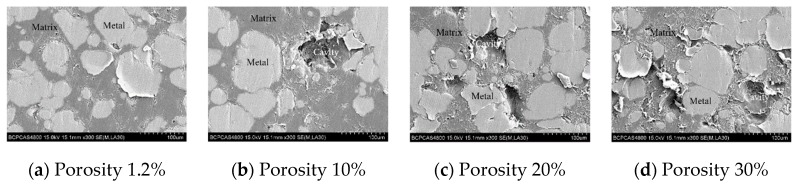
Scanning electron microscope of PTFE/Al with different porosity.

It can be seen from Table 2 that the mass difference of the PTFE/Al sample after sintering was basically the same as that of the additive, and it can be considered that the polypropylene (PP), initially mixed into the material, escaped. The theoretically calculated porosity is less than 10% of the measured porosity, which also reflects that it is feasible to use polypropylene (PP) to prepare PTFE/Al energetic materials with different porosities. Figure 2 shows scanning electron microscope (SEM) images of PTFE/Al with different porosities.

It can be seen from Figure 2 that the Al particles exhibited higher brightness in the backscattered electron imaging, which was due to the higher atomic number of the Al element relative to the C and F elements. In addition, Al particles were distributed discretely in the PTFE matrix. The cavities in the specimens with different porosities were approximately spherical, and the size was basically the same (this is determined by the particle size of the polypropylene added during sintering), and the number of cavities increased significantly with increasing porosity.

### 2.3. Dynamic Loading Experiment 

**Figure 3:**
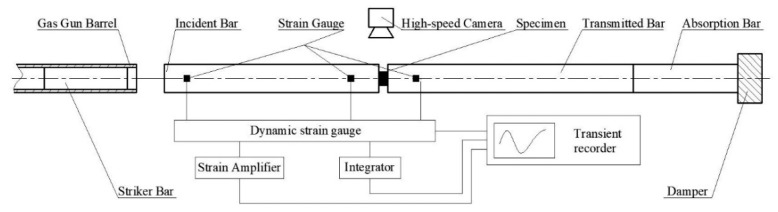
Schematic diagram of the split Hopkinson pressure bar (SHPB) test system.

The split Hopkinson pressure bar (SHPB) test system was used to perform dynamic compression experiments on PTFE/Al active materials with different porosities. The experimental test system is shown in Figure 3. During the experiment, the high-pressure gas chamber drove the striker bar to hit the edge of the incident bar, and the generated stress wave propagated in the incident bar, the specimen, and the transmitted bar. Through the strain-time signal collected by the strain gauge attached to the incident bar and the transmitted bar, the signal was amplified by the dynamic strain gauge. And then, through the signal acquisition of a digital oscilloscope and data processing by a computer, the correlation between internal stress, strain, strain rate, and time could be obtained. A high-speed camera recorded the deformation, failure, and impact-induced chemical reactions of PTFE/Al specimens under impact compression loading.

The steel bar was used for dynamic loading in the experiment. The length of the incident bar and the transmitted bar was 1200 mm, the diameter of the bars was 16 mm, the length of the impact bar was 200 mm, and the size of the specimens was Φ8 × 5 mm. Compared with traditional metal and alloy materials, PTFE/Al energetic materials have lower mechanical strength and mechanical resistance. In order to avoid uneven stress during compression of the material, a rubber shim is placed between the striker bar and the incident bar during impact. Therefore, the rise time of the incident pulse and the dispersion effect of the signal are reduced.

## 3. Results and Discussion 

### 3.1. Impact Response Characteristics of Porous PTFE/Al Energetic Materials 

#### 3.1.1. Impact Reaction of Porous PTFE/Al

**Figure 4:**
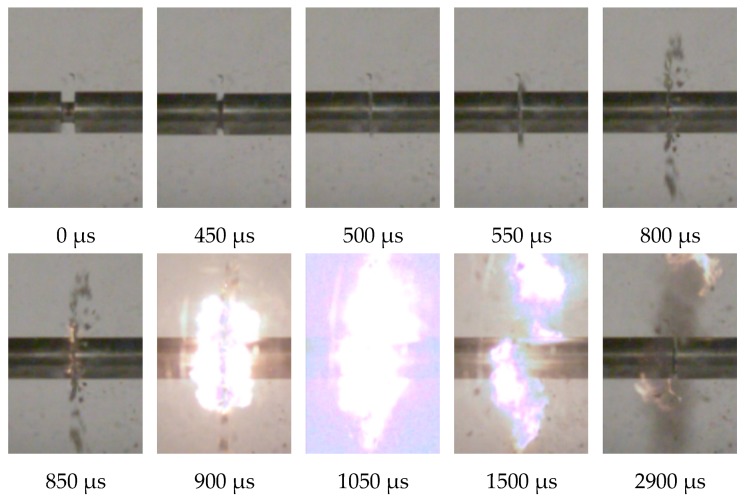
High-speed camera images of dynamic deformation and fragmentation of the PTFE/Al sample with 20% porosity at strain rate 5744 s^−1.^

Figure 4 shows a high-speed camera photograph of PTFE/Al specimens with the porosity of 20% at different times under the strain rate of 5744 s^−1^.

It can be seen from Figure 4 that the specimen was strongly compressed and continuously deformed after the impact, and the strain continued to increase at this time. Assuming that the moment when the incident bar initially struck the specimen was 0, at 450~500 µs the specimen started to fail, and a small amount of debris was spattered out. At 550~800 µs, a large amount of debris was generated probably due to growth and merger of internal cracks [7]. At 800~850 µs, it was seen that the PTFE/Al specimen excitedly reacted and produced a fire. As time went on, the fire gradually strengthened, and then gradually went out. As the interval time from the initial impact of the material sample to the occurrence of flare was the ignition delay time, and the time from the flare generation to the extinction was the reaction duration time, it can be seen that the ignition delay time of the 20% porosity PTFE/Al sample under the loading of 5744 s^−1^ strain rate was 850 µs, and the reaction duration time was 2050 µs.

**Figure 5:**
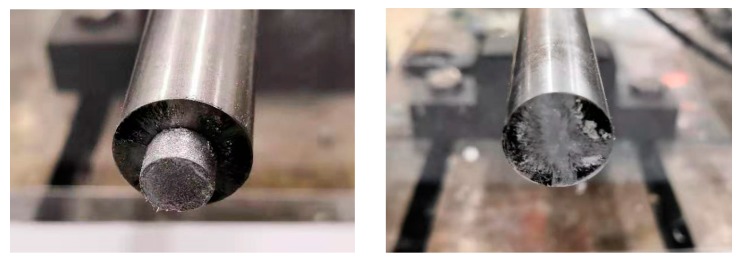
Comparison of specimens before and after SHPB experiment.

**Figure 6:**
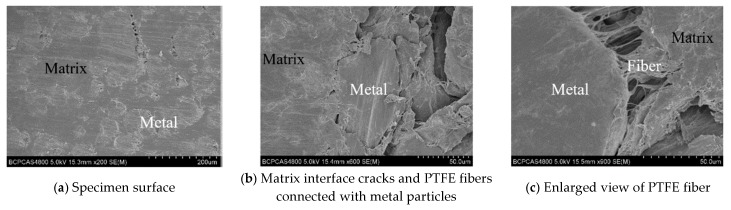
Scanning electron microscope images of the residual specimen.

Figure 5 is a comparison photograph of a 20% porosity PTFE/Al sample before and after the 5744 s^−1^ strain rate loading experiment. It can be seen that the specimen was pressed into a flake. Scanning electron microscopy was used to observe the unreacted residual specimens, and the results are shown in Figure 6.

It can be seen from Figure 6a,b that the Al particles deformed a little after dynamic compression, they no longer maintained the spherical shape, and cracks occured at the junction of the metal particles and the matrix. In Figure 6c, which is partially enlarged, it can be seen that the PTFE fibers in the tensile state appeared at the bond between the matrix and the particles, according to [10], these fibers will further prevent the material from further damage.

#### 3.1.2. Impact Reaction of Porous PTFE/Al 

**Figure 7:**
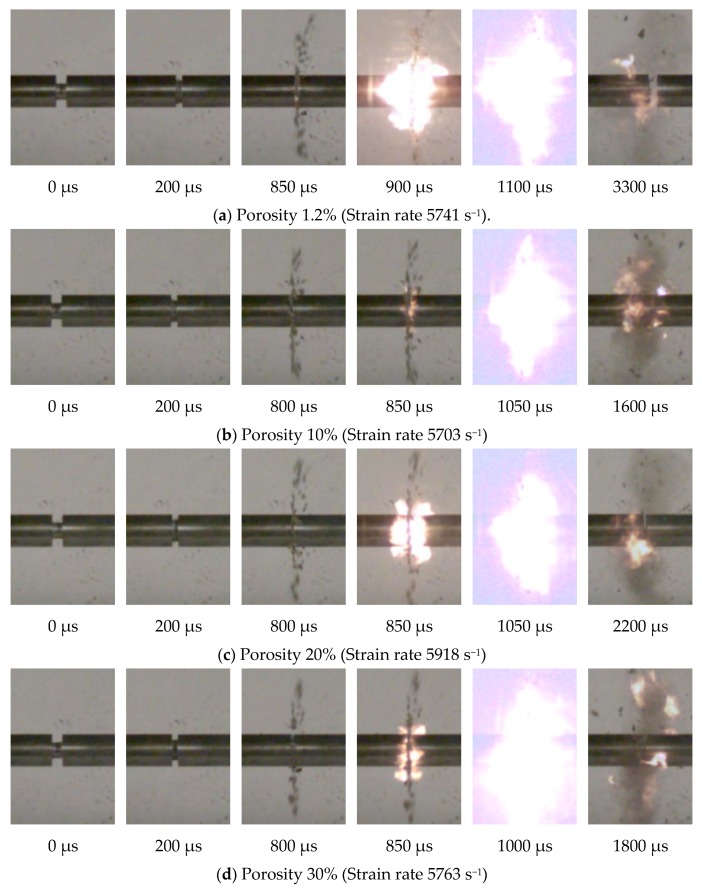
High-speed camera images of PTFE/Al with different porosity at similar strain rates.

Figure 7 shows the high-speed camera photograph of the impact reaction process of PTFE/Al energetic materials with porosities of 1.2%, 10%, 20%, and 30% under strain rates of 5703 to 5918 s^−1^. And the corresponding ignition delay time and reaction duration time are shown in Table 3.

It can be seen from Figure 7 and Table 3 that with the continuous increase of porosity, the ignition delay time was between 850 and 900 µs. According to the analysis, the internal pores of the PTFE/Al specimens will collapse and deform under impact loading, resulting in an increase in the internal energy around the pore size, which will cause a "hot spot" response. Combined with the scanning electron microscope observation results, the increase in porosity was only due to the increase in the number of pores in the material, while the size of the pores was basically unchanged, and the internal energy absorbed by a single hole collapse was the same, so the ignition delay time of PTFE/Al with different porosities remained unchanged. Under the loading strain rate of 5668 s^−1^ to 5918 s^−1^, the PTFE/Al energetic materials with a porosity of 1.2%, 20%, and 30% (except for porosity of 10%), as the porosity increased, the reaction duration time showed a decrease trend, decreasing from 3300 to 1550 µs. According to the analysis, the increase in porosity reduced the mass of the energetic material itself, which resulted in a reduction in the duration of the reaction. The increase in porosity also caused more "hot spots" to be excited inside the material, which may lead to faster material reaction speed and thus reduce the material reaction duration.

In addition, it can be seen from Table 3 that when the loading strain rate was about 5200 s^−1^, the PTFE/Al specimen with a porosity of 10% had a relatively short-term flare, while no flares were observed in other PTFE/Al specimens with the porosity. When the loading strain rate was about 5700 s^−1^, the four kinds of PTFE/Al specimens with different porosities all stimulated chemical reactions and generated flares. It can be seen that there was a critical strain rate for pores containing PTFE/Al energetic materials to react under impact loading conditions. When the strain rate was lower than this value, the reaction would not occur. For the φ8 × 5 mm specimens used in this experiment, the PTFE/Al energetic material with a porosity of 1.2%, 20%, and 30% has a critical strain rate of 5154~5744 s^−1^. The PTFE/Al energetic material with a porosity of 10% had a critical strain rate of 4146 s^−1^ to 5121 s^−1^. It can be concluded that under the load rate of 4146 s^−1^ to 5744 s^−1^, the PTFE/Al energetic materials have an optimal porosity, and the critical strain rate of the material under the optimal porosity is at a low level.

### 3.2. Dynamic Compression Mechanical Properties of Porous PTFE/Al Energetic Materials 

**Figure 8:**
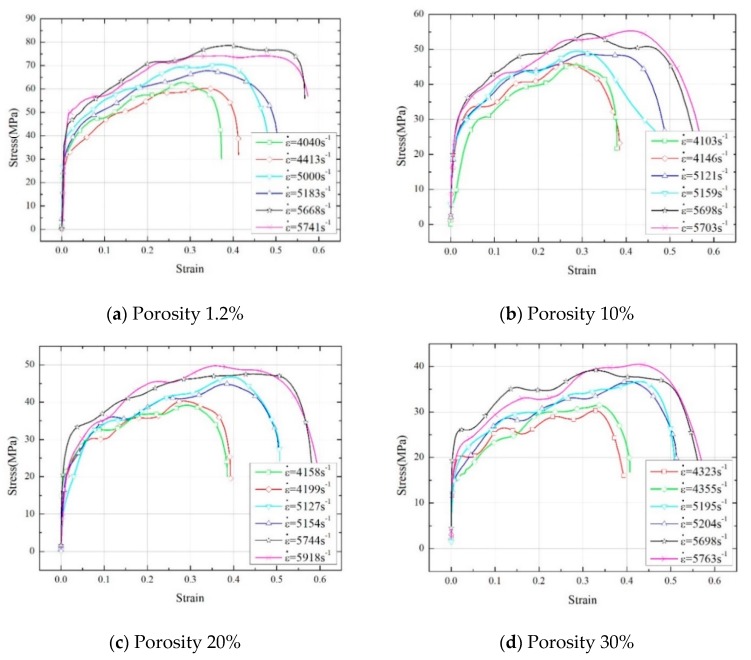
Dynamic compressive stress-strain curve of PTFE/Al with different porosity.

Figure 8 shows the stress-strain curves of four PTFE/Al energetic materials with different porosities under different strain rates. Table 3 shows the experimental data of yield strength, compressive strength, and failure strain of PTFE/Al energetic materials under different strain rates.

It can be seen from Figure 8 and Table 3 that the dynamic compression process of PTFE/Al energetic materials containing pores can be roughly divided into three stages: The elastic stage, the plastic stage, and the failure stage. In the elastic stage, the relationship between stress and strain is approximately linear. As the specimens are further compressed, in the plastic stage, the specimens are deformed and upset macroscopically. The stress in the plastic stage continues to increase with the increase of the strain, which shows a more obvious strain hardening effect. At the failure stage, the specimens are further compressed, the PTFE fibers are continuously broken, and the specimens are damaged and failed. The specimens spattered out residue, and the specimens located between the incident rod and the transmission rod were squeezed into a flake. For a certain porosity PTFE/Al energetic material, as the strain rate increases, the yield strength, compressive strength, and failure strain of the material all show an increasing trend. In addition, at a similar strain rate, as the porosity increases, the yield strength and compressive strength of the material decrease significantly, while the failure strain of the material is almost unchanged. According to the analysis, this is because the existence of pores will cut off the contact between the Al particles and the PTFE matrix, and reduce the contact area. Furthermore, as the porosity increases, the bonding between the particles and the matrix becomes less, resulting in a lower mechanical strength of materials with higher porosity.

### 3.3. Ignition Reaction Mechanism of PTFE / Al with Temperature Rise

Under impact loading, the porous inside the material will collapse and close, and the increase in internal energy caused by the non-uniformity of the material will concentrate on the periphery of the pore, forming a "hot spot", and eventually the "hot spot" will cause an ignition reaction [11]. Field et al. [12] studied the different ignition mechanisms of explosives in local areas, Studies by Field et al. [13] show that although crack tip heating can generate hot spots, its energy effect is slower. Winter and Field [14] show that hot spots are formed in plastically deformed band structures larger than 1 μm in size. They propose that deformation of adiabatic shear bands due to compression and heat generated by internal friction are a potential hot spot mechanism. However, the literature [13] indicates that adiabatic shear is suitable for the formation of hot spots in propellants at low temperatures. Kornfeld and Suvorov [15] find that the collapse of the pores caused the material to ignite. And the pore impact collapse mechanism is suitable for the case of high impact velocity and low yield stress [16]. Due to the variety of holes shape, number, and position, it is very complicated to study the hole collapse and temperature rise under impact loading. Based on the viscoplastic hole collapse temperature rise model proposed by Carroll [17], the melting effect of the medium surrounding the hole was introduced [18], and a temperature rise effect model containing PTFE/Al was used. The assumptions are as follows:

(1) The flow field near the hole is spherically symmetric;
(2) The viscoplastic region and the melting region are dense and incompressible;
(3) Approximate shock loading process with constant pressure P at the outer boundary of the cell;
(4) The PTFE/Al composite material melts at the melting point of PTFE, and the temperature rise near the hole is related to the radius.

The initial porosity *α* of the cell is:

(1) $$\alpha =\frac{\rho_{T}-\rho }{\rho_{T}}=\frac{a^{3}}{b^{3}}$$

where *a* and *b* are the radius of the hole and cell at time *t*, *ρ*_T_ is the theoretical density of the material, and *ρ* is the measured density of the material.

The hole of cell collapse dynamics equation is described by [19]:

(2) $$-a\rho_{T}\ddot{a}=\frac{P-P_{g}}{1-Z}+\rho_{T}{\dot{a}}^{2}\left(2-\frac{1}{2}\frac{1-Z^{4}}{1-Z}\right)+\frac{2}{1-Z}\int_{a}^{b}\left[-Y(P_{T},T)+6\mu (P_{T},T)\frac{a^{2}\dot{a}}{r^{3}}\right]\frac{dr}{r}$$

The initial conditions of the equation are: $a(0)=a_{0},\;\dot{a}(0)=0$

where, $Z=\frac{a}{b}=\frac{a}{\sqrt[{3}]{a^{3}+b_{0}^{3}-a_{0}^{3}}}$, $\dot{a}$ and $\ddot{a}$ are the hole radius velocity and acceleration, *a*_0_ and *b*_0_ are the hole and cell radius at the initial time, and *r* is the value of the calculated point distance with the center of the sphere as the origin. *P* is the pressure applied on the outer surface of the cell. *P*_g_ is the pressure of the adiabatic compressed gas in the holes, and its change law takes the following form:

(3) $$P_{g}=P_{g0}{\left(\frac{a_{0}}{a}\right)}^{3\gamma }$$

*P*_g0_ is the initial gas pressure in the holes, the standard atmospheric pressure *P*_g0_ = 101kPa, *γ* is the adiabatic index, *γ* = 1.4. *Y*(*P*_T_,*T*) and *μ*(*P*_T_,*T*) are the yield stress and viscosity, respectively. Due to the melting effect, *Y*(*P*_T_,*T*) and *μ*(*P*_T_,*T*) cannot be taken as constants, they must be related to the pressure distribution *P*_T_ = (*r*,*t*) and temperature distribution *T* = (*r*,*t*) in the cell spherical shell. Therefore, they can be written as *Y* = (*r*,*t*) and *μ* = (*r*,*t*). The melting point temperature *T*_m_ (for PTFE/Al mixtures at 600 K), the yield stress *Y* = (*r*,*t*) and viscosity *μ* = (*r*,*t*) are as follows:

(4) $$Y(P_{T},T)=\left\{ \begin{array}{ll} Y_{0} & T<T_{m}-30\\ Y_{0}(T_{m}-T)/30 & T_{m}-30<T<T_{m}\\ 0 & T>T_{m} \end{array}\right.$$

(5) $$\mu (P_{T},T)=\left\{ \begin{array}{ll} \mu_{0} & T<T_{m}\\ \mu_{0}\exp(\frac{E_{m}}{T}-\frac{E_{m}}{T_{m}}) & T\geq T_{m} \end{array}\right.$$

where, *T*_m_ is the melting point of the medium under normal pressure, and *E*_m_ is the activation temperature of the viscous fluid. To simplify the problem, the dependence of viscosity on pressure is ignored in the formula.

**Figure 9:**
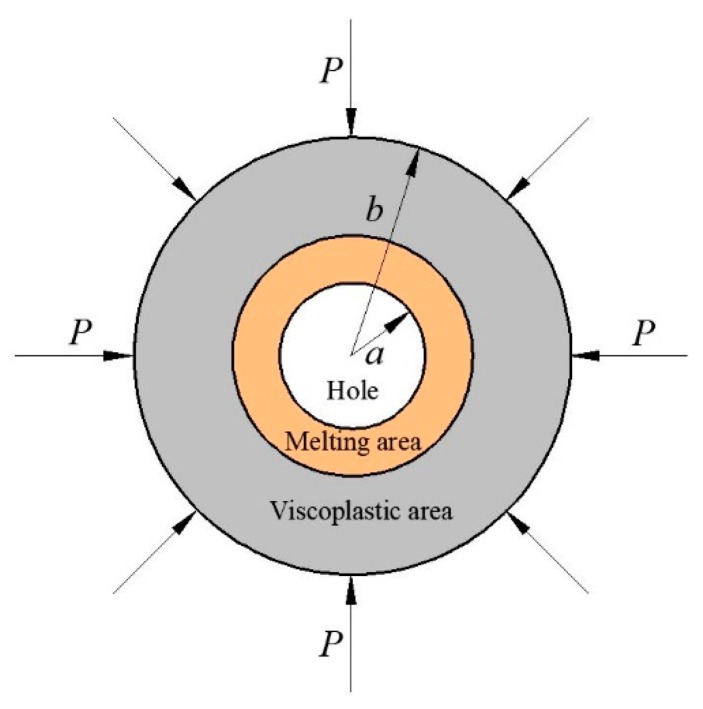
One-dimensional viscoplastic hole collapse model considering the melting effect.

The temperature rise caused by the collapse of the inner diameter of the hole can be expressed as [18]:

(6) $$\rho C_{v}\dot{T}=12\eta \left(\frac{u^{2}}{r^{2}}\right)+2Y\left(\frac{\left|u\right|}{r}\right)$$

where, $u=\frac{\dot{a}a^{2}}{r^{2}}$, *C*_v_ is the heat capacity coefficient, *Y* is the yield strength, the unit is MPa, *η* is the viscosity coefficient, the unit is MPa·μs, $\dot{T}$ is the heating rate of the hole diameter, the derivative of temperature to time between hole and melting area (Figure 9), the unit is K·μs^−1^.

By solving Equations (2) and (6) simultaneously, when the PTFE/Al energetic material containing pores is subjected to external pressure, the change in the hole diameter of the internal pores and the change in temperature rise caused by it can be obtained.

Based on the above impact temperature rise analysis model, calculations are performed on the PTFE/Al energetic materials with a porosity of 10%, 20%, and 30%, the initial calculation parameters of the energetic materials are shown in Table 4 [20,21].

**Figure 10:**
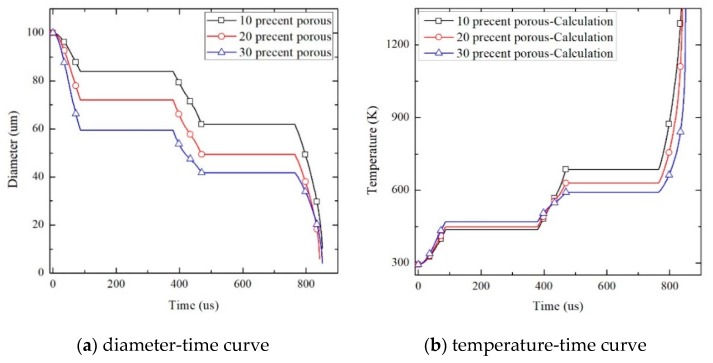
PTFE/Al time-diameter, time-diameter rate, and time-temperature rise curve of different porosity.

Figure 10 shows the pore diameter and temperature change curves of pores and temperature in PTFE/Al energetic materials with 10%, 20%, and 30% porosity under cyclic pressure after data processing.

As can be seen from Figure 10a, the pore diameter of PTFE/Al specimens with porosities of 10%, 20%, and 30% gradually decreases with time, and the medium near the pores is accelerated to the final stage of pore collapse. At higher speeds, the pore radius decreases rapidly. The value of the rate of change in the porosity of the specimen with a higher porosity first reaches the maximum, and the macroscopic performance is that the material is easily compressed.

It can be seen from Figure 10b that before 500 µs, the temperature of the specimen with higher porosity rises faster, it is considered that this is mainly because the greater the change in the inner diameter of the pores with higher porosity, and the temperature rise of the material is mainly determined by $\dot{a}$, the hole radius velocity in the first term on the right side of the polynomial in Equation (6), between 500 and 800 µs, the temperature of the specimen with higher porosity increases slowly. It is considered that this is due to the smaller rate of change in the pores in the specimen with higher porosity during this period, the temperature rise effect of the material is gradually affected by the yield strength *Y* of the second term on the right side of the polynomial in Equation (6). The quasi-static compression experiment also shows that the specimen with higher porosity has lower yield strength. In addition, the specimens with a porosity of 10% given in Table 3 can react at a strain rate of 5121 s^−1^ to 5159 s^−1^, but the specimens with 20% and 30% porosity can react at a strain rate of 5127 s^−1^ to 5204 s^−1^, which confirmed the correctness of the theoretical analysis from the side.

## 4. Conclusions

In this study, four PTFE/Al energetic materials with different porosities were prepared by adding additives, the split Hopkinson pressure bar (SHPB) experimental system was used to study the dynamic mechanical properties of PTFE/Al energetic materials with different porosities and the influence of porosity on the impact response characteristics of materials, based on the one-dimensional viscoplastic hole collapse model, the impact temperature rise analysis model including melting effects was established, and corresponding analysis calculations were performed. The specific conclusions are as follows:

(1) Under dynamic compressive loading conditions, PTFE/Al energetic materials exhibit strain rate effects. At the same strain rate, as the porosity increases from 1.2% to 30%, the yield strength of the material decreases from 29.4~49.8 MPa to 14.6~26.0 MPa, and the compressive strength of the material decreases from 60.3~78.6 MPa to 30.2~40.5 MPa. Failure strains at strain rates of 4040~4413 s^−1^, 5000~5204 s^−1^, 5668~5918 s^−1^ are about 0.31, 0.39, 0.48, respectively.
(2) With the increase of porosity, the ignition delay time of the material is basically unchanged, while the reaction duration generally becomes gradually shorter. When the strain rate is 5600~6000 s^−1^, the ignition delay time is basically maintained between 850 and 900 μs. When the porosity is increased from 1.2% to 30%, the reaction duration is reduced from 3300 to 1550 μs, which is reduced by about 39.6%.
(3) There is a critical strain rate for the reaction of PTFE/Al energetic materials. When the strain rate is lower than this value, the reaction will not occur. The material response has the optimal porosity with the lowest critical strain rate, the optimal porosity of the high aluminum content PTFE/Al energetic material (50/50 mass ratio, size of specimens Φ8 × 5 mm) with a porosity of 1.2%, 10%, 20%, and 30% measured by experiments is 10%.

## Figures and Tables

**Table 1:** Term description.

Properties	Particle Size	Density	Softening Point	Melting Point
Value	100 μm	0.9 g/cm^3^	155 °C	165 °C
Manufacturer	Zhongliansuhua Company, Dongguan, China			

**Table 2:** Parameters of PTFE/Al with different porosity.

PTFE/Al	Masses before Sintering (g)	Masses after Sintering (g)	Balance Masses (g)	Masses of Additives (g)	Theoretical Porosity (%)	Measured Porosity (%)	Error (%)
Specimen 1	17.75	17.73	0.02	0	1.2	1.1	8.3
Specimen 2	17.88	17.03	0.85	0.9	10	10.85	8.5
Specimen 3	17.95	16.12	1.83	1.88	20	21.23	6.2
Specimen 4	17.82	14.71	3.11	3.24	30	32.86	9.5

**Table 3:** Dynamic mechanical parameters of PTFE/Al energetic materials.

Material Properties	Strain Rate (s^−1^)	Yield Strength (MPa)	Compressive Strength (MPa)	Failure Strain	Ignition Delay Time (µs)	Reaction Duration Time (µs)
PTFE/Al (Porosity 1.2%)	4040	29.4	62.8	0.28	Unreacted	-
	4413	32.2	60.3	0.34	Unreacted	-
	5000	38.8	70.5	0.37	Unreacted	-
	5183	36.7	67.8	0.34	Unreacted	-
	5668	45.0	78.6	0.51	900	2250
	5741	49.8	74.1	0.52	850	3300
PTFE/Al (Porosity 10%)	4103	27.9	45.6	0.34	Unreacted	-
	4146	33.2	46.0	0.32	Unreacted	-
	5121	28.9,	48.7	0.31	850	250
	5159	28.0	49.5	0.32	900	100
	5698	36.2	54.6	0.45	850	1800
	5703	32.7	55.3	0.44	850	1600
PTFE/Al (Porosity 20%)	4158	20.3	39.2	0.29	Unreacted	-
	4199	26.4	40.3	0.29	Unreacted	-
	5127	31.3	46.8	0.40	Unreacted	-
	5154	23.2	44.9	0.38	Unreacted	-
	5744	32.8	47.6	0.51	850	2050
	5918	32.4	49.8	0.49	850	2200
PTFE/Al (Porosity 30%)	4323	19.9	30.2	0.33	Unreacted	-
	4355	14.6	31.5	0.33	Unreacted	-
	5195	21.7	36.6	0.43	Unreacted	-
	5204	15.7	36.7	0.4	Unreacted	-
	5698	26.0	39.2	0.49	850	1550
	5763	23.1	40.5	0.45	850	1800

**Table 4:** Properties of PTFE/Al used in calculations [20,21].

PTFE/Al Parameter	Value		Parameter	Value
Yield stress *Y* (MPa)	Porosity 10%	17.70	Viscosity *μ* (Pa·s)	1010
	Porosity 20%	15.97	Melting point temperature *T*_m_ (K)	600
	Porosity 30%	12.62	Activation temperature *E*_m_ (K)	3880 *
Theoretical density *ρ*_T_ (Kg/m^3^)	2.42		Heat capacity coefficient *C*_v_ (J/g/k)	1.59
			Hole initial diameter *a*_0_ (μm)	100

* Limited by conditions, the 50/50 mass ratio of PTFE/Al activation temperature is taken from reference [21].
